# Effect of Citric Acid on Swelling Resistance and Physicochemical Properties of Post-Crosslinked Electrospun Polyvinyl Alcohol Fibrous Membrane

**DOI:** 10.3390/polym15071738

**Published:** 2023-03-31

**Authors:** Ssu-Meng Huang, Shih-Ming Liu, Hua-Yi Tseng, Wen-Cheng Chen

**Affiliations:** 1 Advanced Medical Devices and Composites Laboratory, Department of Fiber and Composite Materials, Feng Chia University, Taichung 407, Taiwan; 2 Department of Fragrance and Cosmetic Science, College of Pharmacy, Kaohsiung Medical University, Kaohsiung 807, Taiwan; 3 Dental Medical Devices and Materials Research Center, College of Dental Medicine, Kaohsiung Medical University, Kaohsiung 807, Taiwan

**Keywords:** electrospinning, membrane, polyvinyl alcohol (PVA), crosslinking, citric acid (CA), biocompatibility

## Abstract

A series of electrospun polyvinyl alcohol (PVA) fiber membranes were crosslinked with citric acid (CA) at concentrations of 10, 20, and 30 wt.% (designated as CA10, CA20, and CA30). The effects of CA on the chemical structure, mechanical strength, swelling resistance, and cytotoxicity of the crosslinked PVA fibrous membranes were investigated. Infrared spectroscopy indicated the enhanced esterification of carboxyl and hydroxyl groups between CA and PVA. The modulus and strength of the electrospun PVA membrane increased due to the crosslinking between CA and PVA. The crosslinking of the PVA fiber matrix with CA increased the PVA binding point, thereby increasing the swelling resistance and modulus; however, the concentration of CA used was limited. Results showed that the water absorption of the PVA membranes decreased from 6.58 ± 0.04 g/g for CA10 to 3.56 ± 3.33 g/g for CA20 and 2.85 ± 0.40 g/g for CA30 with increasing CA. The water absorption remained unchanged after the membrane was soaked for a period of time, so no significant difference was found in the water absorption capacity of the same group after immersion from 1 h to 3 d. The tensile strength increased from 20.52 MPa of CA10 to 22.09 MPa of CA20. With an increased amount of CA used for crosslinking, the tensile strength and modulus of CA30 decreased to 11.48 and 13.94 MPa, respectively. Our study also showed that CA was not toxic to L929 cell viability when used for fiber crosslinking at less than 20 wt.% PVA, meaning it may be a good candidate as a support layer for guided tissue engineering.

## 1. Introduction

Nanofiber composites and membranes have increased in practical applications due to the widespread use of sub-micron to nano-scale fibers [1]. Electrospinning is more commonly used to fabricate micro and nanofibers compared with other conventional processes, such as phase separation, interfacial polymerization, self-assembly, wet spinning, and drawing [2,3,4,5,6,7]. These fibers have many applications, such as reinforcement phase in composites, personal protective textiles, filtration, biomedical devices, electrical and optical devices, nano-sensors, etc. [8,9,10,11,12].

The extracellular matrix (ECM) is primarily a network-supporting structure composed of a variety of biological proteins, polysaccharides, and other components [12,13,14]. The ECM regulates a multitude of cellular processes, including migration, wound healing, and differentiation, which are critical in tissue regeneration. The interconnected network facilitates the diffusion of nutrients and wastes, while the hierarchical pore structure is essential for cell seeding and adhesion. In addition to biomimetic mechanical strength, biocompatibility, and biodegradability, porous 3D scaffolds, films, or membranes should have a fibrous mesh structure for tissue engineering [15]. Biodegradable polymers with controlled lifetimes are promising alternatives to nondegradable polymers. Most importantly, biodegradable polymers have received increasing attention due to their versatility and application potential, especially in the environment and biomedical fields.

Polyvinyl alcohol (PVA) is a typical polyhydroxy polar polymer with comprehensive properties, such as biocompatibility and excellent barrier properties, making it an ideal choice for various guided tissue regeneration (GTR) applications. PVA is a representative water-soluble polymer hydrogel that is more biodegradable than other biopolymers [16,17]. PVA has excellent network structure-forming, emulsifying, and adhesive properties. Furthermore, PVA is suitable for electrospinning without cytotoxic organic solvents due to its water-soluble properties [12,18]. Membranes act as a barrier for GTR therapy [19]; as such, the strength and swelling resistance of the membrane need to be considered in a dental procedure that typically uses a barrier membrane to guide the growth of new bone and gum tissue. However, membranes made from a single biopolymer are highly sensitive to human environmental conditions and typically have low mechanical strength, poor resistance to degradation, and low resistance to swelling in contact with body fluids [20]. Chemical crosslinking is an effective strategy used to impart desired properties to membranes composed of single biopolymers, especially in terms of structural stability [18].

Citric acid (CA), which contains three carboxylic acid groups, is the main organic acid in citrus fruits. CA has been applied in many fields, such as pharmaceuticals, cosmetics, dietary supplements, and food [20,21]. CA is used as a crosslinking agent in polymers and as fixative in the interwoven structures of biopolymers because its carboxylic acids can react with the hydroxyl, amine, and imine groups of biopolymers [22]. The crosslinking of polymers with CA can provide some pendant-free carboxylic acid groups in the branches of the main structure, which may enhance certain biological properties, such as modulating hydrophilicity, increasing binding sites for bioconjugation, and conferring antibacterial activity [23].

The use of CA to crosslink various biopolymers, such as collagen [13], starch [16], hyaluronic acid [24], chitosan [14], gelatin [25,26], and PVA [18,20,27], has been discussed in the literature. However, few investigations have been conducted on using CA to crosslink PVA membranes through electrospinning and the effect of different membranes on the resulting properties and crosslinking interactions [27,28,29]. Moreover, large residual amounts of CA may have negative effects, especially in biomedical applications [12,28,29]. Therefore, this study aimed to develop electrospun PVA membranes through crosslinking CA for biological applications. A series of electrospun PVA hydrogel fibers were prepared to determine the optimal groups and were crosslinked with CA for electrospinning and thermal crosslinking. The effects of CA on morphology, functional groups, and mechanical properties related to swelling resistance and cytotoxicity of fibrous membranes were characterized.

## 2. Materials and Methods

### 2.1. Materials and Membrane Preparations

Polyvinyl alcohol (PVA, (C_2_H_4_O)_n_, Mw: 200,000–240,000 g/mole, white to cream powder, density = 1.3 g/cm^3^, melting point = 230–240 °C) was supplied by Beijing Guoren Yikang Technology Co., Ltd., Beijing, China. Pure pharmaceutical-grade citric acid 1-hydrate (CA, USP, BP, Ph. Eur., JP) was obtained commercially (Panreac Química, SA, Barcelona, Spain). Double-distilled water (ddH_2_O) was used to prepare the gel before electrospinning.

Different amounts of PVA were added to ddH_2_O to form 6, 8, and 10 wt.% PVA hydrogels to select the best concentration for electrospinning. For example, 8 g of PVA was dissolved in 10 mL of ddH_2_O at 100 °C and vigorously stirred at 300 rpm using a magnetic stirrer for 2 h. The solution was slowly cooled to room temperature, and the hydrogel was ready for electrospinning. Homogeneous 8 wt.% PVA was prepared using the dissolution for crosslinking with CA. The amount of 8 wt.% PVA in ddH_2_O was fixed, and 10 wt.%, 20 wt.%, and 30 wt.% of CA relative to PVA were added to verify the effects of CA. The hydrogels were stirred for 2 h at 300 rpm to prepare CA10, CA20, and CA30 solutions. The solutions were slowly cooled to room temperature under stirring for electrospinning. The equipment consisted of a rack-mounted high-voltage DC power supply (AU-60R1-LC, Matsusada Precision, Shiga, Japan) and a syringe pump (YSP-101, YMC Co., Ltd., Kyoto, Japan). For electrospinning, the distance from the 18-gauge blunt-ended metallic needle tip to the collecting plate was 14 cm, the forward speed of the solution was 0.5 mL/h, and the voltage was 15 kV. The electrospun fibrous membranes prepared were placed in an oven at 150 °C for 1 h to crosslink with CA.

### 2.2. Characterization

The surface morphology and structural composition of the membranes were analyzed by an optical microscope (OM, CK, Olympus, Tokyo, Japan) and a scanning electron microscope (SEM, S-3400N, Hitachi, Tokyo, Japan) in a high-vacuum node operating at a voltage of 20 kV, and a Fourier transform infrared spectrometer (FTIR, Nicolet iS5, Thermo Fisher Scientific, Waltham, MA, USA) collecting in the absorbance mode at a wavelength range between 500 and 4000 cm^−1^.

### 2.3. Swelling Characterization 

The membrane was cut into 2 cm × 2 cm pieces and weighed to obtain dry weight (W*_dm_*). The sample was placed into a 15 mL centrifuge tube with 5 mL of ddH_2_O, and placed in a constant-temperature water bath at 37 °C at regular intervals (1, 2, 4, and 8 h, then 1, 2, and 3 d). The surface was wiped with a saturated sponge to remove excess water. The wet membrane sample was weighed (W*_sm_*). The swelling ratio of the crosslinked PVA membrane was obtained using the following equation [16,21,30]:

Swelling ratio (g/g) = (W*_sm_* − W*_dm_*)/W*_dm_*

Twelve replicates (*n* = 12) were measured, and the average was recorded. Membrane swelling tests were also performed in phosphate-buffered saline (PBS; Gibco, Thermo Fisher Scientific Inc., Waltham, MA, USA) at pH 7.4 using the same procedure to verify the morphological changes of the membranes immersed in ddH_2_O and PBS. 

### 2.4. Mechanical Testing

According to the ASTM D882 specification for tensile tests, a PVA fibrous membrane of uniform thickness was selected. The anisotropically electrospun membrane was cut into dumbbell-shaped specimens with a mean thickness of 0.04 ± 0.01 μm [31,32]. The mechanical properties of the post-crosslinked PVA membranes with different concentrations of CA were tested on a universal testing machine (HT-2402, Hung Ta, Taichung, Taiwan) at an elongation rate of 5 mm/min. The change in tensile stress versus strain (SS curve) was recorded until the specimen failed. Peak strength, strain change at failure, and tensile modulus at which elastic deformation occurred were calculated. 

### 2.5. Cytotoxicity

Mouse fibroblasts (L929) were selected for cytotoxicity tests according to the specifications of ISO 10993-5. The cells were cultured in Minimum Essential Medium Alpha (Gibco, Thermo Fisher Scientific Inc., Waltham, MA, USA) containing 10% horse serum, antibiotics (penicillin/streptomycin, P/S), and sodium bicarbonate.

For the quantitative cytotoxicity test, a fibrous membrane with a thickness of less than 0.5 mm was immersed in a cell culture medium with a ratio of 6 cm^2^/mL at 37 °C for 24 h to prepare the sample extract. The control groups included the following: (i) control: cultured with medium to simulate the culture process of normal cells; (ii) positive control: dimethyl sulfoxide (DMSO, Sigma–Aldrich, St. Louis, MO, USA) was mixed with medium to prepare a concentration of 15% DMSO; (iii) negative control: high-density polyethylene (HDPE, Sigma–Aldrich, St. Louis, MO, USA) was used to confirm the effectiveness of sterilization. In brief, 100 µL of medium and L929 cells at 1 × 10^4^ cells/well were transplanted into a 96-well microtiter plate and incubated in a 5% CO_2_ incubator at 37 °C for 24 h. The medium was replaced by the extract, followed by incubation for another 24 h. After removing the extract, 100 µL/well of fresh cell culture medium was added, and 50 µL/well of XTT Cell-Proliferation Assay Kit (Biological Industries, Ltd., Beit-Haemek, Israel) was used. After 4 h of incubation, the sample was tested using an ELISA reader (SPECTROstar Nano, BMG LABTECH, Offenburg, Germany). The XTT-measured absorbance of the mean optical density at 492 nm (OD492) from the ELISA results was proportional to cell viability (i.e., the absorbance of the cells).

Samples for qualitative measurements and controls were prepared in the same way as for cytotoxicity quantification. L929 cells at 1 × 10^5^ cells/well were transplanted in 1000 µL of the medium into 48-well microplates. The cells were cultured for 24 h in an incubator at 37 °C under 5% CO_2_. Cell morphology was observed under an inverted microscope (IVM-3AFL, SAGE VISION Co., Ltd., New Taipei City, Taiwan).

### 2.6. Statistical Analysis

ANOVA was performed using IBM SPSS Statistics version 20 (SPSS Inc., Chicago, IL, USA) to analyze tensile strength, swelling rate, and cell viability. Data were reported as mean ± standard deviation (SD) with a significance level of *p* < 0.05.

## 3. Results and Discussion

### 3.1. Design, Preparation, and Characterization of Electrospun Fibers

**Figure 1:**
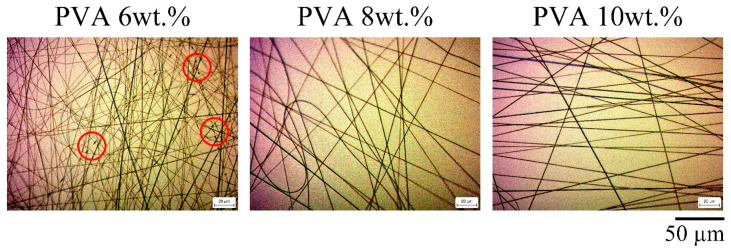
OM images of electrospun fibers at different PVA concentrations and the red circles indicate the location of droplets (magnitude: 500×).

In this work, the PVA gel has a large number of hydroxyl groups, which enable strong intramolecular and intermolecular force interactions and result in high viscosity during electrospinning. The addition of CA did not affect the basic properties of the electrospinning process before heating. Homogeneous but varying concentrations of PVA were dissolved in ddH_2_O for electrospinning (Figure 1). The polymers collected in the 6 wt.% PVA group were mostly smooth fibers but showed a small number of droplets. When the concentration of the PVA solution was increased to 8 wt.% and 10 wt.%, the fiber surface was smooth, and no droplets were generated. However, the operation time for 10 wt.% PVA was longer, so 8 wt.% PVA was used for subsequent experiments.

**Figure 2:**
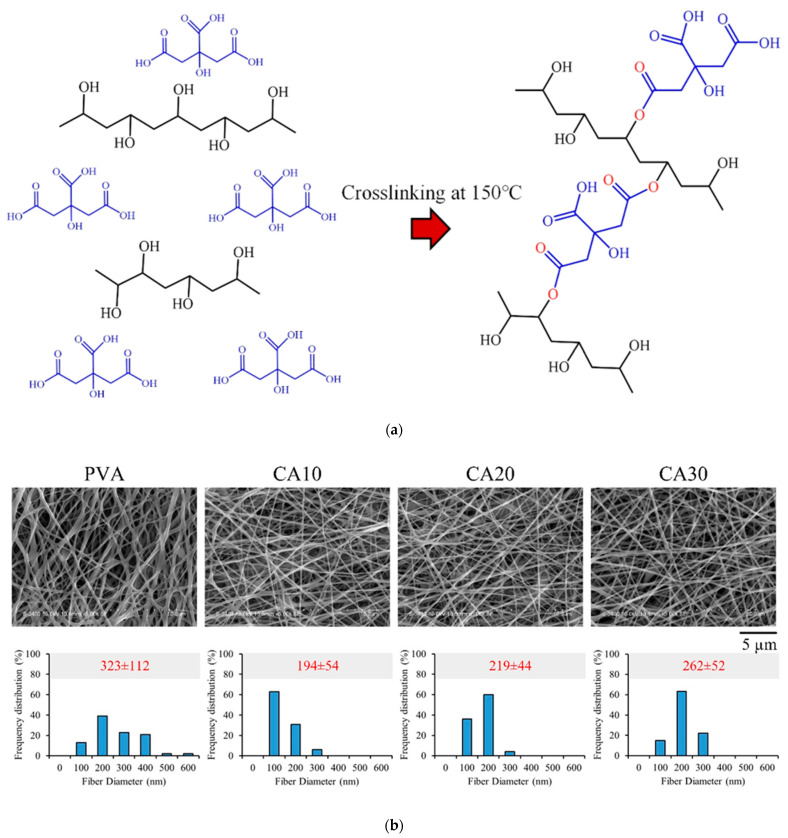
(**a**) Schematic diagram of crosslinking polyvinyl alcohol (PVA, black molecular formula) with citric acid (CA, blue molecular formula). (**b**) SEM images of PVA membranes without crosslinking and crosslinked with CA of different concentrations (magnitude: 5000×).

The homogeneous hydrogel of PVA and CA was dissolved in ddH_2_O to form a fibrous structure supported by PVA, which was then crosslinked by CA to reinforce the fibrous structure (Figure 2a). The carboxyl group of CA underwent esterification with the hydroxyl group of PVA, resulting in crosslinking of PVA during heating and dehydration. Some of the carboxylic acid groups of CA did not sufficiently react with PVA during the crosslinking process, so free carboxylic acids on the membrane enhanced the hydrophilicity of the membrane surface and provided binding sites to modulate cellular processes [21]. 

The SEM images of electrospun PVA crosslinked with different CA concentrations are shown in Figure 2b. The nanofibers without crosslinked CAs showed larger diameters than those with CA during electrospinning. CA is a polar molecule and acts as a plasticizer in PVA [20], resulting in higher viscosity during high-voltage electrospinning and thus, a narrower fiber diameter distribution compared to uncrosslinked PVA treated by electro-optic.

### 3.2. Tensile Strength of Membranes

**Figure 3:**
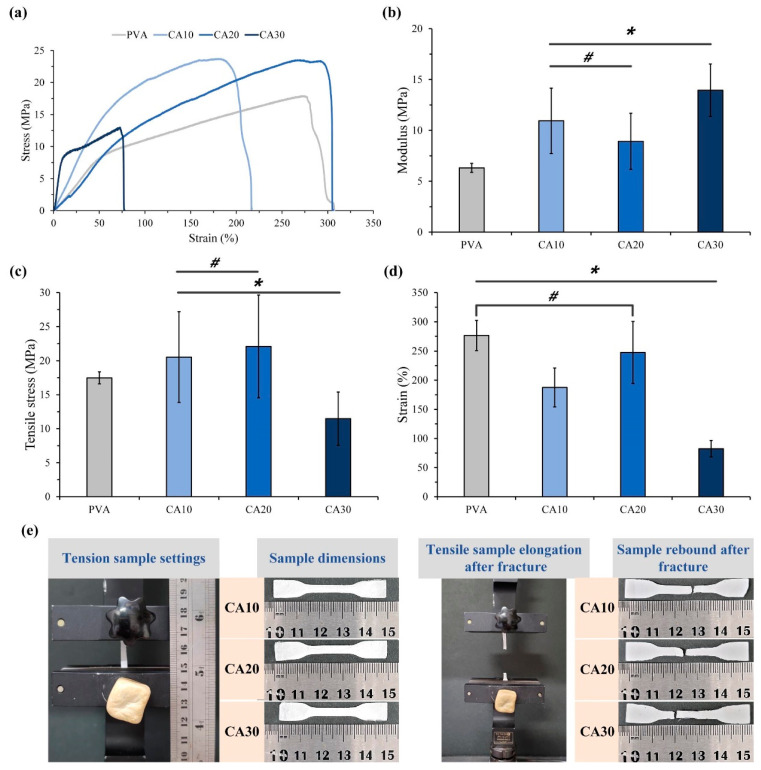
Tensile test results for PVA without crosslinks and CA10, CA20, and CA30 of PVA post-crosslinked membranes (*n* = 6; vertical error bars represent uncertainty (standard deviation) in parameter CA, * indicates significant difference at *p* < 0.05 and # means the two groups were not statistically significant at *p* > 0.05): (**a**) typical tensile stress–strain curves, (**b**) measured modulus, (**c**) values of tensile strength, (**d**) failure strain, and (**e**) sample dimensions, sample morphologies, and samples before and after tensile fracture (images left to right).

A barrier GTR membrane requires a certain tensile strength to maintain the integrity of the hydrogel when guiding and regulating the direction of cell attachment, proliferation, and differentiation. Therefore, the mechanical properties of CA-crosslinked PVA were evaluated through the tensile test. As shown in Figure 3a, CA20 exhibited higher strength and resilience than pure PVA, CA10, and CA30 membranes. The modulus of CA20 was lower than those of CA10 and CA30 but was not significantly different from that of the controlled PVA. These findings are further confirmed in Figure 3b–d, which reveal significant improvement in the mechanical properties in the CA20 group. The CA-crosslinked PVA hydrogel had a denser and more complete structure; as such, the content of CA significantly contributed to the improvement of the mechanical properties of the electrospun PVA membrane, which manifests fiber shrinkage and narrower fiber diameter distribution in the SEM results (Figure 2a,b). Lin et al. (2020) reported that the tensile and compressive properties of bioactive glass crosslinked PVA hydrogel were enhanced with increasing PVA content [33]. Crosslinking plays an important role in improving the mechanical properties of the PVA network structure, and an optimal amount of crosslinking agent should be used. We analyzed the elastic modulus of the hydrogel, and the results are consistent with the stress–strain curves (Figure 3b). The sample rebounded due to shape recovery, leading to the coupling between the crosslinked shape memory and Poisson’s ratio effect (Figure 3e). As a result, the shortening in the compression direction was greater than the expansion in the tension direction. However, the stretch deformation was less pronounced than the deformation perpendicular to the stretch axis due to the crosslink rebound effect [31]. In the presence of the crosslinking agent CA, the PVA membranes can undergo intermolecular and intramolecular crosslinking during the heating process, and the number of crosslinking points between PVA molecules increases, thereby making the membranes tougher. In this study, CA20 membranes with proper PVA crosslinking and improved mechanical properties were obtained instead of the maximum crosslinking conditions of CA30.

### 3.3. FTIR Spectra

**Figure 4:**
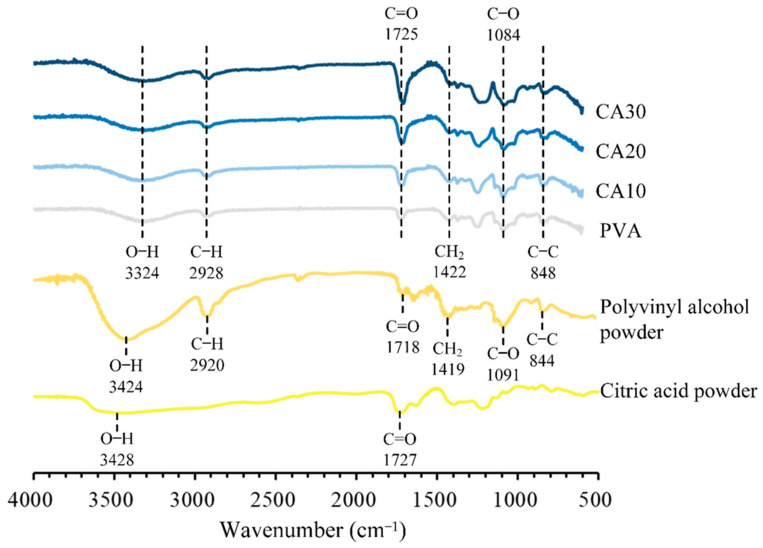
FTIR spectra of raw materials, PVA membranes without crosslinking, and PVA membranes crosslinked with CA of different concentrations.

The infrared spectra of PVA crosslinked with different CA concentrations are shown in Figure 4. All major absorption bands related to hydroxyl and acetate groups were observed [34]. The characteristic bands of PVA are the stretching band of O–H at 3424 cm^−1^, the vibrational band of C–H at 2920 cm^−1^, which refers to the stretching of C–H from alkyl groups, and the stretching band of C=O at 1718 cm^−1^, which is due to the acetate group remaining from PVA. The band at 1419 cm^−1^ is the bending vibration of C–H_2_, the band at 1091 cm^−1^ is the absorption of C–O, and the band at 844 cm^−1^ is the absorption peak of C–C. The characteristic bands of CA are the wide band of O–H stretching vibrations at 3428 cm^−1^ and the –COOH stretching of C=O at 1727 cm^−1^ [35]. 

The spectra (CA10, CA20, and CA30) of PVA were compared before and after crosslinking with CA. The characteristic absorption of PVA before and after crosslinking did not disappear or increase. In the stretching vibration at 1725 cm^−1^, the intensity of the C=O band increased with the addition of CA, which is evidence of intermolecular interaction between PVA and CA. The increased strength can be attributed to the degree of esterification of PVA and CA crosslinking and the increase in carboxylic acid groups in the crosslinked network. In addition, the absorbance of the C=O groups changed due to the formation of some hydrogen bonds between the C=O and –OH groups in the PVA molecules due to the heat treatment with the crosslinker CA.

### 3.4. Swelling Resistance of Fibrous Membranes

**Figure 5:**
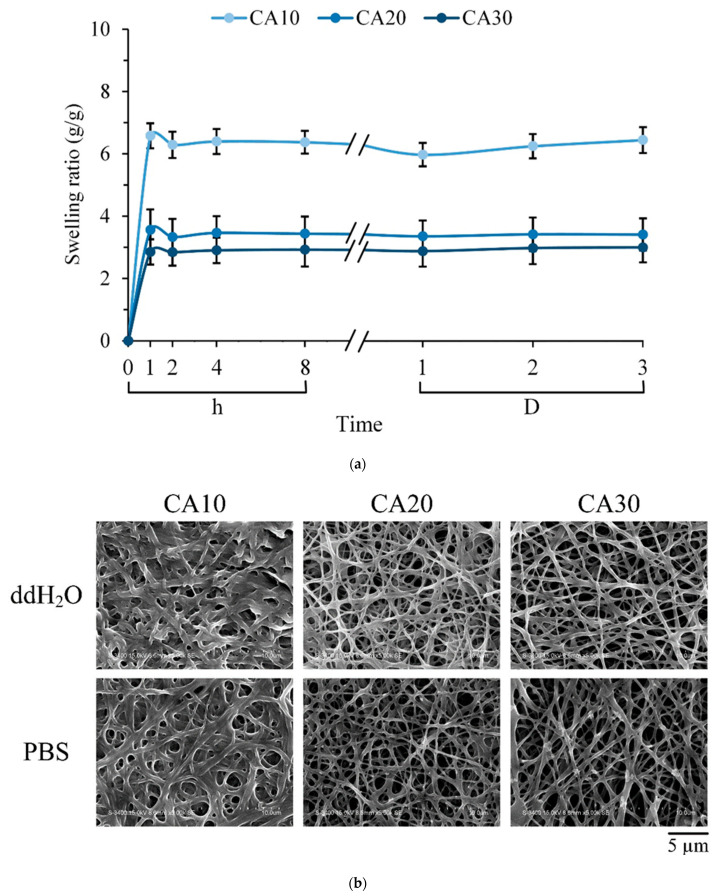
(**a**) Swelling behavior measured by weight change of PVA membranes crosslinked with CA of different concentrations soaked in ddH_2_O for 3 days (*n* = 12; vertical error bars represent uncertainty (standard deviation) in parameter CA). (**b**) Morphological changes of different membranes after soaking in ddH_2_O and PBS for 3 days.

For GTR membranes, the swelling rate is critical to prevent early decay before tissue regeneration. Figure 5a shows the weight change of PVA membranes crosslinked with different concentrations of CA soaked in water for 3 d. Each group reached the highest water absorption rate after soaking for 1 h, and no significant change in weight was observed until 3 d. This finding proves that CA was successfully crosslinked to the PVA hydrogel. The water absorption weight of CA10 was higher than those of CA20 and CA30. CA10 had a higher fiber swelling rate than CA20 and CA30, in addition to water absorption in the pores between fibers.

Due to its high-water solubility, adhesion, permeability, and film-forming properties, PVA is widely used as a membrane. In Figure 5b, the fibers of each group maintained their shape after soaking in ddH_2_O and PBS for 3 d, while the fibers of CA10 swelled significantly compared with those of CA20 and CA30. Accordingly, PVA crosslinked by heating, in the presence of crosslinker CA, showed excellent swelling resistance. Crosslinking PVA with CA can improve the problem of easy hydrolysis of PVA [16,36], and the change in CA concentration can affect the fiber shape after immersion. Given that CA has a multi-carboxyl structure, the carboxyl groups in CA and the hydroxyl groups in PVA can undergo esterification, thereby improving water resistance [20,37]. Although the anti-swelling ability was enhanced, the elastic modulus of CA30 (Figure 3a) was also largely increased, resulting in a decrease in resilience since the CA30 membrane contained relatively few hydroxyl groups in PVA. 

The swelling decreased with the degree of esterification and crosslinking, as evident in the spectral results (Figure 4). The decrease in the swelling degree of the crosslinker with increasing CA concentration has limitations due to the competition between CA as a plasticizer and a crosslinker between PVA molecules. In summary, the crosslinking of PVA with 20 wt.% CA was not degraded after soaking and maintained the fiber shape. Therefore, CA20 was selected as the optimal group and used for subsequent biocompatibility tests.

### 3.5. In Vitro Biocompatibility

**Figure 6:**
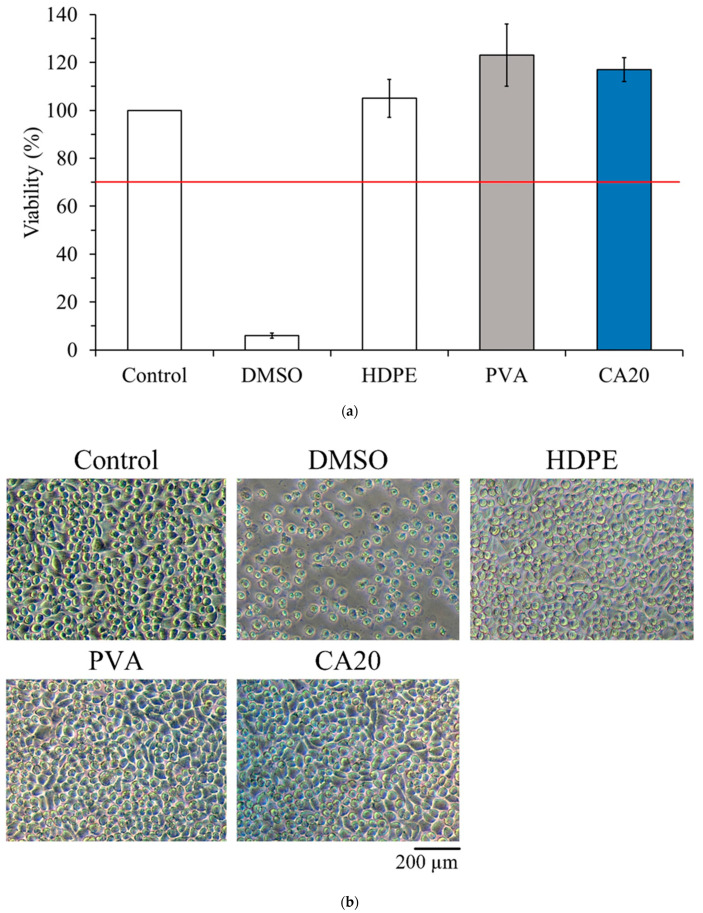
Cytotoxicity of L929 cells cultured in sample extracts according to ISO 10993-5 specification: (**a**) quantitative measurements (*n* = 3), and (**b**) qualitative cell morphology observation.

Hydrogel membranes for biomedical applications must have good biosafety and biocompatibility. Figure 6a shows the cytotoxicity of the PVA membrane crosslinked with CA in L929 fibroblasts cultured for 1 d. Testing was conducted based on the ISO10993-5 standard; if the cell survival rate is higher than 70%, then the sample does not have cytotoxicity. The survival rate of the cells in the CA20 group was higher than 70%, indicating the lack of cytotoxicity. This finding proves that the addition of CA20 for crosslinking of the PVA membrane did not cause cytotoxicity. Furthermore, based on the qualitative results of the cells (Figure 6b), no cytotoxicity was recorded.

Post-crosslinked PVA membranes can be combined with other membranes to improve mechanical properties. PVA membranes can be effective as a physical barrier against postoperative adhesion [38,39,40], although their role in promoting cellular behavior remains controversial. For example, by increasing the ratio of PVA in the PVA/collagen composite hydrogel, the viability of human gingival fibroblasts decreased [41], suggesting that PVA hydrogel has the potential to resist gingival tissue ingrowth. By contrast, Kim et al. [42] studied the attachment and serial proliferation rates of human prostate epithelial cells on electrospun polycaprolactone mats containing PVA, and the introduction of PVA increased the proliferation rate of the cells. In another study, fibroblasts were grown on PVA-based electrospun biomembranes; the results showed that PVA supported cell growth and proliferation [43,44]. In conclusion, PVA was chosen in combination with other materials as a barrier or facilitator layer for use as a GTR membrane to provide mechanical support and prevent rapid epithelial attachment or promote target cell proliferation.

## 4. Conclusions

This study demonstrated the use of CA as a crosslinker to synthesize PVA hydrogel membranes. PVA hydrogel membranes (8 wt.%) were prepared by electrospinning and dehydrated after heating to catalyze the ester reaction. With increasing CA concentration (CA10, CA20, CA30), the fiber diameter first decreased and then slightly increased, and the trend was opposite to the swelling rate. The PVA fiber membrane chemically crosslinked with 20 wt.% CA did not degrade, and the fiber shape was better maintained than CA10 after soaking. The strength and strain properties of CA20 were superior to the other groups, especially compared with CA30. In vitro cell experiments proved that the CA20-crosslinked PVA membrane has excellent biocompatibility. Our study proposes a method for the preparation of PVA hydrogel membranes with good cytocompatibility and degradation resistance. Further in vivo studies should be conducted on the application of CA post-crosslinked PVA membrane to GTR.

## Data Availability

The data presented in this study are available on request from the corresponding author.
